# Surveillance for *Borrelia* spp. in Upland Game Birds in Pennsylvania, USA

**DOI:** 10.3390/vetsci7030082

**Published:** 2020-06-30

**Authors:** Christopher A. Cleveland, Liandrie Swanepoel, Justin D. Brown, Mary Jo Casalena, Lisa Williams, Michael J. Yabsley

**Affiliations:** 1 Southeastern Cooperative Wildlife Disease Study, Department of Population Health, College of Veterinary Medicine, University of Georgia, Athens, GA 30601, USA; liandrie.swanepoel25@uga.edu (L.S.); myabsley@uga.edu (M.J.Y.); 2 Department of Veterinary and Biomedical Sciences, Pennsylvania State University, University Park, PA 16802, USA; jubrown42@gmail.com; 3 Pennsylvania Game Commission, 2001 Elmerton Avenue, Harrisburg, PA 17110, USA; mcasalena@pa.gov (M.J.C.); liswilliam@pa.gov (L.W.)

**Keywords:** Borrelia, game birds, Lyme disease, ticks, tick-borne pathogens, zoonoses

## Abstract

The *Borrelia* genus contains two major clades, the Lyme borreliosis group, which includes the causative agents of Lyme disease/borreliosis (*B. burgdorferi* sensu stricto and other related *B. burgdorferi* sensu lato genospecies), and the relapsing fever borreliosis group (*B. hermsii, B. turicatae*, and *B. parkeri*). Other unclassified reptile- and echidna-associated *Borrelia* spp. (i.e., *B. turcica* and ‘*Candidatus* Borrelia tachyglossi’, respectively) do not belong in either of these two groups. In North America, *Borrelia* spp. from both of the major clades are important pathogens of veterinary and public health concern. Lyme disease is of particular interest because the incidence in the northeastern United States continues to increase in both dogs and humans. Birds have a potentially important role in the ecology of *Borrelia* species because they are hosts for numerous tick vectors and competent hosts for various *Borrelia* spp. Our goal was to investigate the prevalence of *Borrelia* spp. in four free-living species of upland game birds in Pennsylvania, USA including wild turkey (*Meleagris gallopavo*), ruffed grouse (*Bonasa umbellus*), ring-necked pheasants (*Phasianus colchicus*), and American woodcock (*Scolopax minor*). We tested 205 tissue samples (bone marrow and/or spleen samples) from 169 individuals for *Borrelia* using a flagellin gene (*flab*) nested PCR, which amplifies all *Borrelia* species. We detected *Borrelia* DNA in 12% (24/205) of samples, the highest prevalence was in wild turkeys (16%; 5/31), followed by ruffed grouse (13%; 16/126) and American woodcock (3%; 1/35). All pheasants (n = 13) were negative. We sequenced amplicons from all positive game birds and all were *B. burgdorferi* sensu stricto. Our results support previous work indicating that certain species of upland game birds are commonly infected with *Borrelia* species, but unlike previous studies, we did not find any relapsing fever borreliae.

## 1. Introduction

Spirochetes in the genus *Borrelia* are transmitted by ticks and use mammalian reservoirs, most often a rodent [1]. *Borrelia* infections of reservoir hosts are often asymptomatic; however, when transmitted to some aberrant hosts (e.g., humans and dogs), infection may result in various disease syndromes. In the United States, the more important diseases include Lyme disease (caused by the *Borrelia burgdorferi* sensu stricto (s.s.) and the recently described *Borrelia mayonii*) and several tick-borne relapsing fevers caused by *B. hermsii, B. turicatae,* and *B. parkeri* (transmitted by with soft ticks) and *B. miyamotoi* (transmitted by *Ixodes scapularis*) [1,2]. The ecology of *B. burgdorferi* s.s. in the United States is well-researched but there is still much to learn about natural history of this important human and animal pathogen as well as other *Borrelia* spp. [3,4,5]. In the eastern United States, the predominant vector of *B. burgdorferi* s.s. is the black-legged tick (*Ixodes scapularis*) [6], while the western black-legged tick (*Ixodes pacificus*) serves as the vector on the Pacific coast. Lyme disease is most prevalent in the Northeast and upper Midwest, but a large number of cases are also reported in the mid-Atlantic and Pacific coast regions [7].

The risk of Lyme disease in the northeastern United States continues to increase, particularly in Pennsylvania, which has prompted interest in wildlife hosts of vectors [7,8,9]. Birds are of particular interest as some species undergo long-range movements, many have been identified as hosts for *Ixodes* species, and they may act as reservoirs for *Borrelia* spp. [10,11]. Upland game birds can act as hosts to both ticks and *Borrelia* species [12,13]. Previous surveillance studies on wild turkeys from Tennessee and California reported a relatively high prevalence of relapsing fever *Borrelia* spp. and a low prevalence of *B. burgdorferi* sensu lato [13,14]. To date, there has been little work investigating the prevalence of *Borrelia* species in North American game birds and none within the northeastern United States [12,13,15].

The goal of this study was to investigate the prevalence of infection with *Borrelia* species among four species of upland game birds (wild turkey (*Meleagris gallopavo*), ruffed grouse (*Bonasa umbellus*), ring-necked pheasants (*Phasianus colchicus*), and American woodcock (*Scolopax minor*) from Pennsylvania, USA.

## 2. Materials and Methods

During 2014–2017, using hunter harvested animals we opportunistically collected bone marrow and spleen samples from wild upland game birds from Pennsylvania. All game birds were free-living and not from captive-propagated sources. Tissue samples were collected and processed as previously reported [16]. Bone marrow samples were collected from the tarsometatarsus (ring-necked pheasants and wild turkeys) or tibiotarsus (ruffed grouse and American woodcock) using sterile bone rongeurs, scalpel blade, and forceps. Spleen samples, when available, were collected using sterile scalpel blades and forceps. Collection of these samples was reviewed by and approved by UGA’s Institutional Animal Care and Use Committee (A2013 07-003).

Tissue samples were stored at −80 °C until DNA extractions were performed using a commercial kit (Qiagen DNeasy Blood and Tissue Kits Germantown, Maryland) following the manufacturer’s instructions. Extracted samples were screened for the presence of *Borrelia* spp. using nested PCR targeting the *Borrelia* flagellin gene (*flab*) as described [17,18]. The primary reaction contained 5 μL of template DNA and was carried out using 1.0 μM concentration (each) of the primers FlaLL (5′-ACATATTCAGATGCAGACAGAGGT-3′) and FlaRL (5′-GCAATCATAGCCATTGCAGATTGT-3′). The nested reaction contained 1 μL of primary PCR product, and 1.0 μM concentration (each) of the primers FlaLS (5′-AACAGCTGAAGAGCTTGGAATG-3′) and FlaRS (5′-CTTTGATCACTTATCATTCTAATAGC-3′). Cycling conditions for both reactions included initial denaturation for 3-min at 95 °C, followed by 40 cycles of 1-min denaturation at 95 °C, 1-min annealing at 55 °C, and 1-min extension at 75 °C. Amplicons were visualized in a 0.8% agarose gel, and positive bands (~350-bp) were excised and extracted using a Qiagen Gel Extraction kit (Qiagen Germantown, MD, USA). Negative and positive controls (from sequence confirmed *B. lonestari* DNA) were used to validate PCR amplification. Purified amplicons from samples were submitted for bidirectional Sanger sequencing at the Georgia Genomics and Bioinformatics Core (Athens, GA, USA). Chromatograms were analyzed using Geneious (Biomatters Limited–Version 11.1.5, Auckland, New Zealand). Sequences were aligned in MEGA (Molecular Evolutionary Genetics Analysis-Version 7.0.21, State College, PA, USA), visually analyzed and compared to sequences in GenBank.

## 3. Results

We tested a total of 205 tissue samples from 169 individual upland game birds including 28 American woodcock (19 bone marrow, 16 spleen), 13 ring-necked pheasants (11 bone marrow, 2 spleen), 97 ruffed grouse (69 bone marrow, 57 spleen), and 31 wild turkeys (17 bone marrow, 14 spleen) (Table 1). Some birds yielded a sample of each bone marrow and spleen, whereas others did not (Table 1).

Overall, 12% (24/205) of the samples were positive for *B. burgdorferi* and no other *Borrelia* species were detected (Table 1). Wild turkeys had the highest prevalence of *B. burgdorferi* of the four species of birds sampled, 16%, (5/31), but *B. burgdorferi* was only detected from bone marrow samples of these individuals, at 29% (5/17). Conversely, 6% (1/16) of woodcock spleen samples were positive, whereas the bone marrow samples were all negative (*n* = 19). Finally, ruffed grouse had positives detected in both bone marrow (16%; 11/69) and spleen (12%; 7/57) (Table 1). None of the ring-necked pheasant bone marrow samples were positive for *Borrelia* spp. Sequence results from positive amplicons were compared to accessioned GenBank species, and resulted in 100% identity agreement to *B. burgdorferi* s.s.

## 4. Discussion

In the United States, Lyme disease is an important vector-borne disease, accounting for 82% of tick-borne associated bacterial infections [19], and has spurred increased interest in other pathogenic *Borrelia* species (e.g., *B. miyamotoi*) [20]. Although several studies have investigated the prevalence of *Borrelia* spp. in *I. scapularis*, few studies have looked at infection in possible avifauna hosts [21,22,23,24,25]. Pennsylvania ranks as one of the leading states for the number of human Lyme disease cases and the number of dogs testing positive for antibodies to *B. burgdorferi* [8,19,26] (www.capcvet.org/maps). Since 1992, Lyme disease has been an increasing disease concern in Pennsylvania as evidenced by the increase in *B. burgdorferi* seroprevalence in dogs, reported cases of Lyme disease in humans, and geographic distribution of cases throughout the Commonwealth [7,8,26,27].

*Borrelia* spp. infections have been noted in upland game birds from several states during previous studies. In Tennessee, wild turkey and migratory waterfowl were screened for *Borrelia* spp., with samples collected close to and distant from Tennessee National Wildlife Refuge (TNWR), a location known for being a stop-over for migratory birds [12]. Prevalence of *B. burgdorferi* was highest in resident wild turkeys sampled closest to the migration routes, whereas *B. lonestari* prevalence was highest in wild turkeys farther away from the TNWR and associated flyways. Another study in Tennessee found 58% (35/60) of wild turkeys were positive for *B. miyamotoi* [13]. Interestingly, in that study, none of the wild turkey blood samples were PCR positive for *B. burgdorferi* or *B. lonestari*, although some studies have reported relapsing fever *Borrelia* spp. that were not detected in the current study. Although no relapsing fever *Borrelia* (i.e., *B. lonestari* or *B. miyamotoi*) were detected in any of the game birds included in our study, the primer set we used is known to amplify several relapsing fever *Borrelia* spp. (including *B. miyamotoi)* as demonstrated by studies in our lab (*B. lonestari* in deer and deer ticks [28], *B. turicatae* in dogs [5], and a novel *Borrelia* sp. in penguins [29] and by other groups [30,31].

The prevalence of *B. burgdorferi* differed between the game bird species surveyed in this study. A potential explanation for this difference in Pennsylvania is the utilization of different habitat types by each avian species, and what species of ticks (and their densities) are thought to co-occur in these areas. For example, wild turkeys utilize habitats including mature coniferous and mature hardwood forest stands, agricultural lands, and a varying degree of interspersion of mature forests and field edges [32,33,34]. Ring-necked pheasants inhabit grasslands and pastoral settings, with some use of scrub/shrub wetlands and stream corridors, which is also habitat shared by woodcock [35,36]. Both woodcock and ruffed grouse are early successional species, found primarily in young forest stands with a high density of woody stems [37]. As previously noted, ticks were not tested as part of this current study, but the prevalence we detected in these game birds was lower than previous reports of *Borrelia* spp. detected in ticks from Pennsylvania (47.4%) [23].

Recently, a 117-year retrospective analysis was conducted evaluating changes in tick communities in Pennsylvania [38]. The study found that five species of ticks (*Dermacentor variabilis, Ixodes scapularis, I. cookei, Rhipicephalus sanguineus, and Amblyomma americanum*) were predominantly represented in collections from 1900–2017 [38]. Ticks recovered from wildlife made up only 6% (275/4491) of the total submissions, and of those submissions, ticks from avifauna were poorly represented. This poses a challenge to the surveillance of ticks and tick-borne pathogens in the avian hosts of Pennsylvania throughout North America.

## 5. Conclusions

In the face of increasing interactions between wildlife and domestic animals, enhanced surveillance of vector and host species is required to provide a robust understanding of tick-borne pathogens and risk assessment. An integral part of tick-borne disease surveillance is increasing our understanding of the role of wildlife species as hosts for ticks and/or reservoirs of pathogens. Migratory birds can carry ticks and pathogens over great distances while upland game birds can host these ticks and may have exposure to pathogens [12,13,22,39]. On a global scale, our results support additional studies documenting findings of *Borrelia* species in avifauna, and importantly the associated vectors in various locations [40,41,42], highlighting the importance of birds in the transmission, maintenance and geographic spread of tick-borne pathogens. In this study, we reported *B*. *burgdorferi* infection of wild upland game birds in Pennsylvania; however, additional work (tick transmission trials, experimental infections with wild turkeys, assessment of clinical illness, continued field surveillance) is needed to provide a more comprehensive perspective on the role of upland game birds in the ecology of *Borrelia* spp. and other tick-borne pathogens and any potential impact of ticks and tick-borne pathogens on the health and survival of wild game birds.

## Figures and Tables

**Table 1:** Number of individual upland game birds and sample type (bone marrow, spleen) from Pennsylvania and prevalence of *Borrelia burgdorferi* detected.

Species (No. of Individuals Sampled, No. of Birds with both Bone Marrow and Spleen Available)	No. of Positive/No. of Samples Tested		Total Prevalence
	Bone Marrow	Spleen	
American Woodcock *Scolopax minor* (n = 28, 7)	0% (0/19)	6% (1/16)	3% (1/35)
Ring-necked pheasant *Phasianus colchicus* (n = 13, 0)	0% (0/11)	0% (0/2)	0% (0/13)
Ruffed Grouse *Bonasa umbellus* (n = 97, 29)	16% (11/69)	12% (7/57)	14% (18/126) *
Wild Turkey *Meleagris gallopavo* (n = 31, 0)	29% (5/17)	0% (0/14)	16% (5/31)
Total (n = 169)	14% (16/116)	9% (8/89)	12% (24/205)

* 8 birds were positive for *Borrelia* from both bone marrow and spleen samples.
